# Increased Risk of Hereditary Prostate Cancer in Italian Families with Hereditary Breast and Ovarian Cancer Syndrome Harboring Mutations in *BRCA* and in Other Susceptibility Genes

**DOI:** 10.3390/genes13101692

**Published:** 2022-09-21

**Authors:** Giovanna D’Elia, Gemma Caliendo, Maria-Myrsini Tzioni, Luisa Albanese, Luana Passariello, Anna Maria Molinari, Maria Teresa Vietri

**Affiliations:** 1 Unity of Clinical and Molecular Pathology, AOU, University of Campania “Luigi Vanvitelli”, 80138 Naples, Italy; 2 Division of Cellular and Molecular Pathology, Department of Pathology, University of Cambridge, Cambridge CB2 1QP, UK; 3 Department of Precision Medicine, University of Campania “Luigi Vanvitelli”, 80138 Naples, Italy

**Keywords:** hereditary prostate cancer, hereditary breast and ovarian cancer syndrome, *BRCA* genes, susceptibility genes

## Abstract

Hereditary prostate cancer (HPCa) has the highest heritability of any cancer in men. Interestingly, it occurs in several hereditary syndromes, including breast and ovarian cancer (HBOC) and Lynch syndrome (LS). Several gene mutations related to these syndromes have been identified as biomarkers in HPCa. The goal of this study was to screen for germline mutations in susceptibility genes by using a multigene panel, and to subsequently correlate the results with clinical and laboratory parameters. This was undertaken in 180 HBOC families, which included 217 males with prostate cancer (PCa). Mutational analysis was further extended to 104 family members of mutated patients. Screening of HBOC families revealed that 30.5% harbored germline mutations in susceptibility genes, with 21.6% harboring pathogenic variants (PVs) and 8.9% having variants of uncertain significance (VUS). We found PVs at similar frequency in *BRCA1* and *BRCA2* genes (8.8% and 9.4%, respectively), while 0.56% of PVs were present in well-established susceptibility genes *PALB2, TP53* and *RAD51C*. Moreover, 0.56% of monoallelic PVs were present in *MUTYH*, a gene whose function in tumorigenesis in the context of PCa is still unclear. Finally, we reported double heterozygosity (DH) in *BRCA1/2* genes in a single family, and found double mutation (DM) present in *BRCA2* in a separate family. There was no significant difference between the mean age of onset of PCa in HBOC families with or without germline mutations in susceptibility genes, while the mean survival was highest in mutated patients compared to wild type. Furthermore, PCa is the second most recurrent cancer in our cohort, resulting in 18% of cases in both mutated and non-mutated families. Our investigation shows that PVs were located mostly in the 3′ of *BRCA1* and *BRCA2* genes, and in *BRCA2*, most PVs fell in exon 11, suggesting a mutation cluster region relating to risk of HPCa. A total of 65 family members inherited the proband’s mutation; of these, 24 developed cancer, with 41 remaining unaffected.

## 1. Introduction

Prostate cancer (PCa) is a heterogeneous disease, representing one of the major causes of malignancy-related deaths worldwide, with about 1,600,000 cases and 366,000 deaths annually [1]. Men with first-degree male relatives affected by PCa have at least a two-fold risk of developing PCa compared to men without a family history of PCa [2]. Approximately 5–15% of PCa cases are attributable to hereditary factors [3]. Hereditary prostate cancer (HPCa) has the highest heritability of any cancer in men, with tumorigenesis occurring due to the presence of germline mutations transmitted through autosomal dominant inheritance [4]. In comparison to sporadic cases, HPCa shows an early age of onset, an aggressive disease progression and locally advanced stage, with a higher risk of recurrence after surgery.

HPCa arises in several hereditary syndromes, including hereditary breast and ovarian cancer (HBOC) and Lynch syndrome (LS) [5]. Several genes related to these syndromes, such as DNA damage repair (DDR) genes *BRCA1, BRCA2, CHEK2, ATM* and *PALB2*, and DNA mismatch repair (MMR) genes *MLH1, MSH2, MSH6* and *PMS2*, were identified as biomarkers in HPCa. However, mutations in *HOXB13, BRP1, NBS1, RAD51C, RAD51D* and *TP53* genes have been found to confer an increased risk of HPCa [6,7,8,9].

The mutation status of genes involved in HPCa could have potential clinical applications, particularly for the stratification of patients into specific treatment and prognostic groups. Patients who harbor mutations in DDR genes, for example, would likely be more sensitive to a treatment regime that includes PARP inhibitors, whereas the mutational status of MMR genes in metastatic patients could predict their response to immunotherapy [10].

Current guidelines do not adequately inform at what disease stage genetic testing should be performed to identify germline mutations in patients who could potentially benefit from alternative treatment. Moreover, the identification of germline mutations in a patient with HPCa could facilitate the genetic testing of family members at risk of HPCa or other associated cancers.

The goal of the study was to identify germline mutations insusceptibility genes using a multigene panel in HBOC Italian families with at least one case of PCa by correlating clinical and laboratory parameters. Mutational analysis was extended to family members of the mutated patients.

## 2. Materials and Methods

### 2.1. Patients

This study was carried out in accordance with the World Medical Association Helsinki Declaration (1964). Informed consent was obtained from all subjects, and the study was approved and conducted according to the ethical guidelines of the University of Campania “Luigi Vanvitelli” (n.469-23/07/2019). The study was conducted at the U.O.C. Clinical and Molecular Pathology, A.O.U. University of Campania “Luigi Vanvitelli”.

We enrolled180 HBOC families that included 217 males with PCa. The probands were affected with prostate, breast, ovarian, colorectal, pancreatic, endometrial, bladder, gastric cancer, or melanoma (Table 1), with an age range of 28–86 years. All selected patients received genetic counselling. Case and family history were collected, and a pedigree was generated for each family. The patients were selected according to the criteria for HBOC syndrome [11], in which mutations occur in high-penetrance *BRCA*1/2 genes, followed by *PALB2*, and mutations in genes that confer moderate penetrance risk, such as *ATM*, *CHEK2*, *RAD51C, RAD50*, *BRIP1*, *PTEN*, *NBN*, *MRE11A*, *BARD1*, *STK11*, *CDH1*, *MUTYH* and *TP53*. Peripheral blood samples were collected in two test EDTA tubes from all patients. Mutational analysis was extended to 104 family members of 34 mutated patients.

### 2.2. Mutational Analysis

Genomic DNA from blood samples was extracted using the Wizard Genomic DNA purification kit (Promega Corporation, Madison, WI, USA) according to the manufacturer’s instructions.

For mutational analysis, we used the TruSight Sequencing Cancer Panel on a MiSeq platform (Illumina, HCS, Sophia Genetics, Switzerland) that analyzes 16 genes, *BRCA1* (NM_007295), *BRCA2* (NM_000059), *ATM* (NM_000051.4), *CHEK2* (NM_007194), *PALB2* (NM_024675), *RAD51C* (NM_058216), *RAD50* (NM_002878), *BRP1* (NM_001003694.2), *PTEN* (NM_000314.8), *NBN* (NM_002485), *MRE11A* (NM_005591.4), *BARD1* (NM_000465.4), *STK11* (NM_000455.5), *CDH1* (NM_004360), *MUTYH* (NM_001128425.1) and *TP53* (NM 000546). The presence of point mutation was confirmed on the other blood sample by Sanger sequencing, as previously described [12]. Molecular analysis in family members of mutated probands was performed by Sanger sequencing, as previously described [13,14]. The results were analyzed using Mutation Surveyor^®^ software, version 3.24 (Softgenetics, State College, PA, USA).

### 2.3. Genetic Variant Classification

ClinVar and LOVD databases were used for the identification and classification of genetic variants. Genetic variants found were categorized according to criteria developed by International Agency for Research on Cancer recommendations [15] and categorized into five classes: benign (class I), likely benign (class II), variant of uncertain significance (VUS, class III), likely pathogenic (class IV) and pathogenic variants (PVs, class V).

## 3. Results

**Figure 1:**
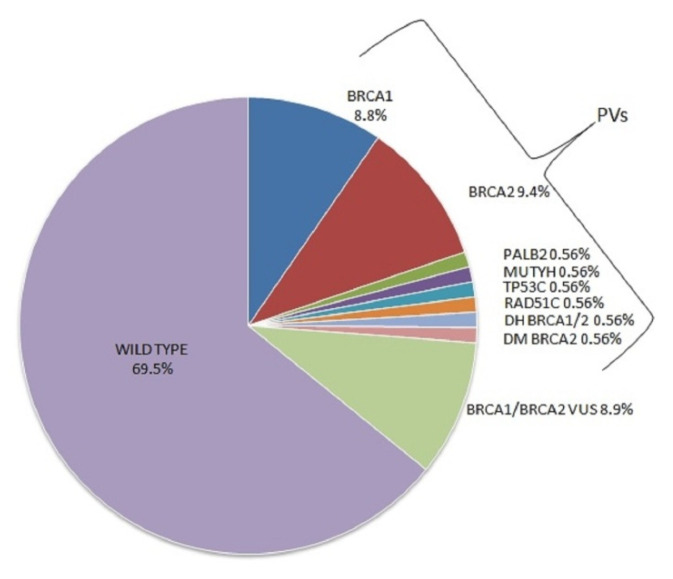
Pathogenic variants and VUS found in HBOC families.

Mutational screening conducted in 180 HBOC families revealed 55 (30.5%) germline mutations in 51 patients. These mutations included 39/180 (21.6%) PV and 16/180 (8.9%) VUS (Figure 1). Moreover, 20/39 (51.3%) of the observed PVs were frameshift, 6/39 (15.4%) intronic variants, 6/39 (15.4%) nonsense, 6/39 (15.4%) missense and 1/39 (2.5%) copy number variation (CNV).

### 3.1. Mutations in DNA Damage Repair Genes

From the families carrying PVs, 16/180 (8.8%) harbored a PV in *BRCA1*, 17/180 (9.4%) in *BRCA2*, 1/180 (0.56%) in *PALB2*,1/180 (0.56%) in *TP53* and 1/180 (0.56%) in *RAD51C*. We also observed double heterozygosity (DH) in *BRCA1/2* genes (0.56%) in a single family, and found double mutations (DM) present in *BRCA2* (0.56%) in a separate family (Figure 1).

The *BRCA1* mutation c.5123C>A(p.Ala1708Glu) was found in three separate families and c.5266dupC (p.Gln1756Profs) in four families; the *BRCA2* mutation c.4133_4136del (p.Thr1378fs) was observed in two families and c.6468_6469delTC (p.Gln2157Ilefs) in three families.

### 3.2. Mutations in Base Excision Repair Gene

**Figure 2:**
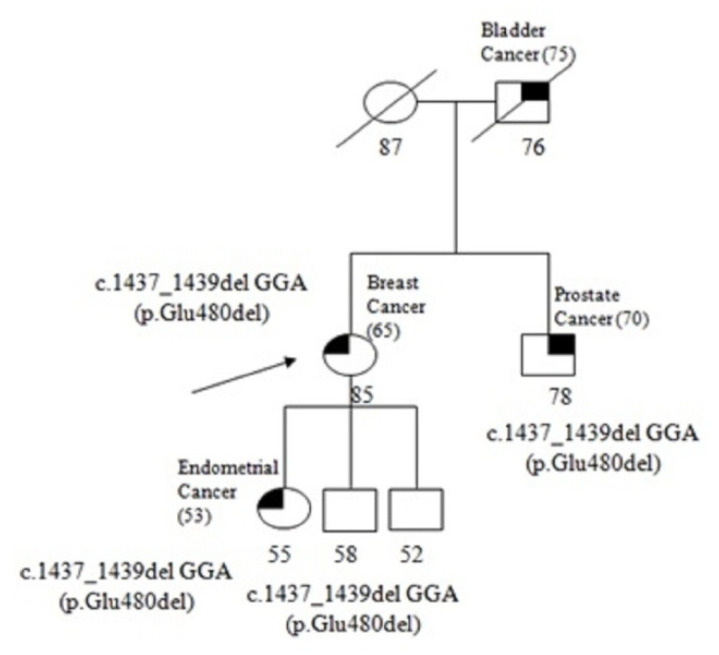
Pedigree of the proband carrying the monoallelic *MUTYH* mutation c.1437_1439del GGA (p.Glu480del). The ages at diagnosis are indicated in brackets.

In the families with observed PVs, 1/180 (0.56%) carried a monoallelic PV in the *MUTYH* gene (Figure 1). This specific mutation consists of a frameshift deletion in exon 14 of the gene (between nucleotides 1437 and 1439), resulting in the introduction of a premature stop codon at amino acid position 480.It was identified in an 85-year-old woman diagnosed with breast cancer at the age of 65. Figure 2 reports the pedigree analysis of the proband; her brother, affected with prostate cancer, inherited the mutation. In addition, the mutational analysis was extended to the daughter, who was diagnosed with endometrial cancer at 55 years of age, and to the unaffected son of 58 years, revealing that the mutation was also present in both the daughter and son.

### 3.3. Genotype–Phenotype Correlation

**Figure 3:**
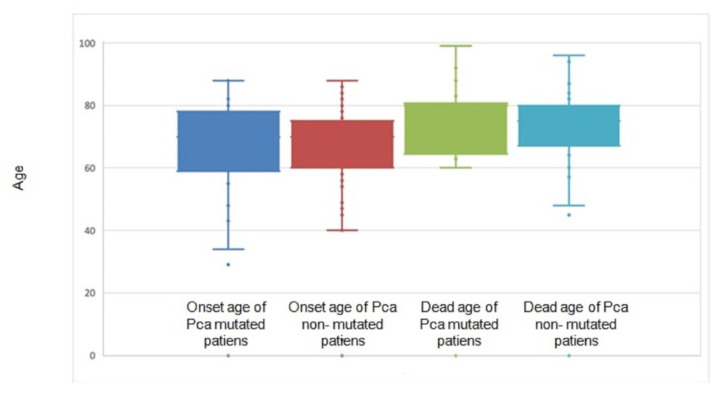
Onset age of 217 PCa patients. HBOC-mutated families included 49 individuals, and non-mutated families 168 individuals. Age of death of 66 PCa patients is also illustrated in this plot.

We observed a mean age of PCa onset of 67.4 years in mutated patients and 68.3 years in non-mutated patients. Of the 217 cases in the cohort, 66 patients died. The mean survival was 8 years and was higher in mutated patients (77.5 years) than in non-mutated patients (74.1 years). Patients who were carriers of VUS were included in the non-mutated patient group (Figure 3)**.**

**Figure 4:**
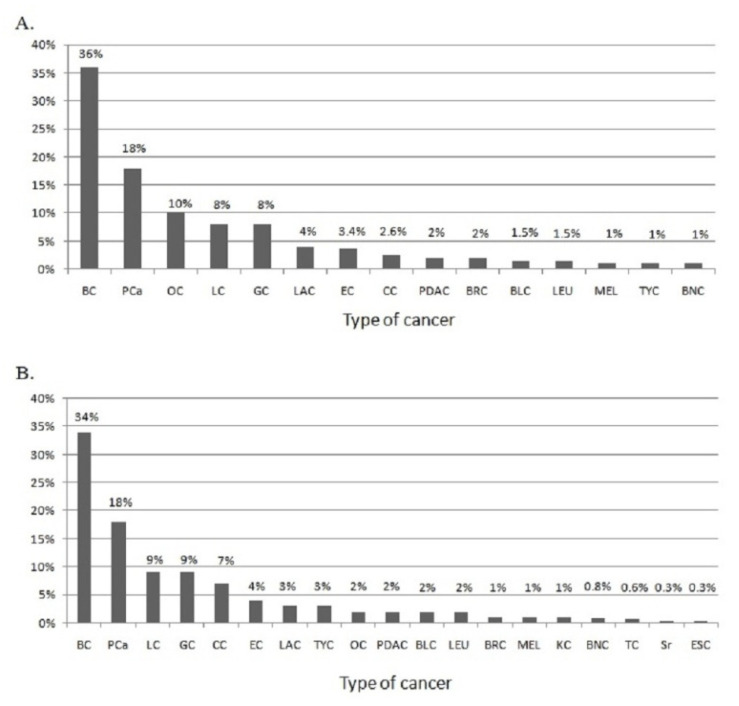
(**A**) Cancer types that occur in HBOC-mutated families. (**B**) Cancer types that occur in HBOC non-mutated families. BC: breast cancer; PCa: prostate cancer; OC: ovarian cancer; LC: lung cancer; GC: gastric cancer; EC: endometrial cancer; LAC: laryngeal cancer; CC: colon cancer; PDAC: pancreatic ductal adenocarcinoma; BRC: brain cancer; BLC: bladder cancer; LEU: leukemia; MEL: melanoma; TYC: thyroid cancer; BNC: bone cancer; KC: kidney cancer; TC: testicular cancer; Sr: sarcoma; ESC: esophageal cancer.

The number and percentage of cancer types that occur in mutated and non-mutated HBOC families are shown in Figure 4. PCa is the second most recurrent cancer, resulting in 18% of cases in both mutated and non-mutated families.

**Figure 5:**
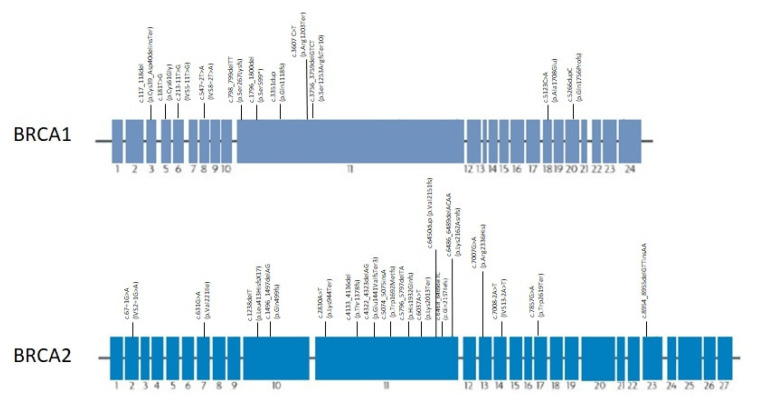
Pathogenic variants found in HBOC families with PCa localized through *BRCA1* and *BRCA2* genes.

Figure 5 illustrates the location of PVs identified in our cohort in both the *BRCA1* and *BRCA2* genes. In both genes, the mutations are located throughout the length of gene, mostly in 3′. In *BRCA2*, most of them fall in exon 11.

Mutational analysis was extended to 104 family members of 30 proband patients harboring mutations in *BRCA1* and/or *BRCA2* genes and four proband patients who were carriers of mutations in *TP53, PALB2, RAD51C* or *MUTYH* genes. This included both cancer-free family members and family members who were diagnosed with cancer. In the remaining eight families with *BRCA* mutations, it was not possible to extend the mutational analysis to family members. The results of the genetic testing are summarized in Table 2. A total of 39 family members only displayed the wild-type gene, while 65 had inherited the proband’s mutation. Moreover, 24 of the 65 individuals who inherited the mutations of the proband developed cancer. The analysis was also conducted in families with DH and DM, showing that these pathogenic variants co-segregate.

## 4. Discussion

The main findings of the present study are as follows. (1) This is one of the few studies focused on Italian patients with hereditary prostate cancer; we have shown pathogenic germline mutations in five susceptibility genes and a monoallelic frameshift mutation in MUTYH, a potentially pathogenic monoallelic mutation whose significance in PCa has not been established. (2) The mutational frequency between *BRCA1* and *BRCA2* genes was similar, and higher than previously reported. (3) We observed frequent PVs in the 3′ of both *BRCA1* and *BRCA2*, and a large number of PVs in *BRCA2* were present in exon 11 of the gene, suggesting a mutation cluster region relating to risk of HPCa. (4) *MUTYH* could be related to overall HPCa risk. (5) The mean age of onset in PCa cases that recur in HBOC families was no different in mutated PCa patients versus non-mutated, while the mean survival was highest in mutated patients compared to wild type. (6) PCa is the second most recurrent cancer from our cohort, resulting in 18% of cases in both mutated and non-mutated families.

We identified mutations in 21.6% of HBOC families; mutations in *BRCA1* and *BRCA2* were found at similar frequency in our cohort. The percentage of mutations found is higher than reported in previous studies that have previously demonstrated that most mutations present were primarily in *BRCA2,* 1.2–5.3%, while *BRCA1* mutations were found in 0.9–1.25% of HPCa patients [16,17,18,19,20,21,22,23,24,25,26]. The difference in mutation prevalence could be influenced by the selection of the patients included; indeed, studies including only HPCa patients report a lower mutation rate than studies including HPCa families. We also identified a family that had DM in both *BRCA1* and *BRCA2* genes, with a separate family harboring two mutations in *BRCA2.* These pathogenic variants co-segregate and do not generate a worse phenotype than a single mutation [27].

Patients who were carriers of genes with VUS, where the variants were not classified as either benign or pathogenic, were difficult to interpret in terms of clinical management. The evaluation of their pathogenic significance needs to be corroborated by further experimental evidence. Co-segregation analysis and in silico analysis may be helpful to improve VUS classification [28,29].

Previous studies proposed that mutations in *BRCA2* exon 11 were closely related to breast/ovarian cancer risk and aggressiveness [30]. Patel et al. hypothesized that the region between c.7914 and 3’ of the *BRCA2* gene is a cluster region associated with an increased risk of developing prostate cancer [31]; however, only one of the mutations found in our patients falls in that region. In the present study, most of the PVs fell in the 3′ of both *BRCA1* and in *BRCA2* genes and in exon 11 of *BRCA2* gene, suggesting a mutation cluster region relating to risk of HPCa. Additional research is expected to confirm this association.

PVs in low-penetrance genes, *PALB2*, *RAD51*C and *TP53,* are present in 0.56% of families. This is in line with previously published studies indicating mutations in other genes in about 0.1–0.2% of cases [19,32]. Despite the rarity of reported *PALB2* alterations, recent findings have supported an increasing role of *PALB2* in PCa, particularly in metastatic cases [33]. *TP53* genetic alterations were reported in 3–12.5% of cases which were considered late events and associated with metastatic castrate-resistant prostate cancer (mCRPC) [34]; however, other studies reported an inactivation of *TP53* at a high frequency in primary as well as metastatic castration-naïve PCa [35].

Biallelic *MUTYH* mutations have been linked to *MUTYH*-associated polyposis syndrome (MAP). In the past few years, monoallelic *MUTYH* PVs have been found in patients with gastric, liver, endometrial, breast, ovarian and pancreatic cancer [36,37], and this has led to several studies investigating the impact of germline monoallelic *MUTYH* PVs in tumorigenesis. We found that 0.56% of families showed a monoallelic frameshift *MUTYH* PV, while a previous study reported *MUTYH* PVs in 2.37% of patients with a family history of prostate cancer [38]. Our results suggest an involvement of monoallelic *MUTYH* mutations in PCa onset; however, additional studies are required.

Mutated and non-mutated patients had a similar mean age of onset, although HPCa showed a trend towards an earlier onset age compared to sporadic cases [39]. No difference has been reported in overall survival between HPCa and sporadic PCa [2]. However, previously, Castro et al. described that BRCA1/2 mutations confer a more aggressive PCa phenotype and lower survival. Additionally, Narod et al. observed a median survival from diagnosis of 4.0 years for men with BRCA2 mutation in antithesis to 8.0 years for men with BRCA1 mutation, a factor that was recommended to be considered for the clinical management of patients [40,41,42]. We found an average survival of 8 years in PCa patients, with a similar average age of death between mutated and non-mutated. This difference could be due to the size of cohort as well as different clinical aspects of the enrolled patients. *BRCA2* germline mutation provided a greater contribution to increased PCa risk compared to *BRCA1*. Carriers of *BRCA2* mutations have an 8.6-foldelevated risk of developing HPCa compared to non-carriers; the relative risk of HPCa developing in *BRCA1* mutation carriers is increased 3.8-fold [43,44]. *BRCA2* pathogenic variants have also been associated with an increased risk of high-grade disease and progression to mCRPC [2]. Here, in HBOC families, PCa occurs in 18% of cancer cases, making it the second most recurrent cancer. The frequency of PCa was equal in both mutated and non-mutated families. Interestingly, there was no significant difference in prostate cancer development between non-mutated HBOC families and HBOC families that harbored mutations in BRCA, PALB2, RAD51C, TP53 and MUTYH genes. This is likely because the categorized “non-mutated” HBOC families could be carriers of pathogenic mutations in genes not yet identified. Screening for mutation in *BRCA1* and *BRCA2* has the potential to help in the stratification of patients to more targeted therapies. Indeed, patients with mutations in the *BRCA* genes have a better response to PARP inhibitors. This led the Food and Drug Administration (FDA) to approve the drug Olaparib for patients with mCRPC who harbor mutation in BRCA1/BRCA2 and/or ATM genes. MMR-mutated patients can benefit from immunotherapy, with the FDA having recently approved PD-L1 inhibitor pembrolizumab (KEYTRUDA) for the treatment of patients with microsatellite instability-high (MSI-H)/MMR-deficient [45,46,47]. 

Furthermore, clinical trials undertaken with prostate cancer patients who are carriers of a mutant *PALB2* have demonstrated a positive response to Olaparib treatments, particularly for patients with resistance to treatment [33,48].

*TP53* mutations have been associated with an unfavorable response to antiandrogens, abiraterone and enzalutamide, although this mechanism remains to be elucidated. Whether and to what extent *TP53* alterations affect the response to the PARP inhibitor Olaparib is currently unclear [35].

Surveillance should be conducted regularly with PSA assay, urological examination and/or magnetic resonance imaging annually, starting from age 41. In our cohort, the 41 unaffected HBOC family members who inherited the proband’s mutation have an increased risk of developing PCa or other related tumors. The potential consequences relating to tumorigenesis could be reduced through the early detection of the cancer. Therefore, all 41 unaffected HBOC family members who are carriers of the proband’s mutations were enrolled in an active surveillance program for PCa and other related tumors in our hospital. Women with *BRCA1*, *BRCA2*, *PALB2* and *RAD51C* mutations were enrolled in a surveillance program for breast, ovarian and colon cancer development, whereas men with *BRCA1*, *BRCA2*, *PALB2* and *RAD51C* mutations were enrolled in a surveillance program targeting breast, prostate, colon, laryngeal and pancreatic cancer.

## 5. Conclusions 

In this study, one of the few studies focused on Italian patients with hereditary prostate cancer, we showed pathogenic germline mutations in five susceptibility genes in HBOC probands. In addition, we founded a monoallelic frameshift mutation in *MUTYH* gene, suggesting an involvement of monoallelic *MUTYH* mutations in PCa onset. Testing family members of mutated patients gives them the opportunity to optin to surveillance programs for HPCa and other HBOC-related tumors, decreasing the potential consequences relating to cancer development through early detection.

## Figures and Tables

**Table 1:** Number and tumor type of HBOC probands.

Syndrome	No. of Families	No. and Tumor Type of Probands	No. of PCa Cases
Hereditary breast and ovarian cancer syndrome (HBOC)	180	154 breast cancers	217
		8 ovarian cancers	
		3 colorectal cancers	
		3 melanomas	
		2 prostate cancers	
		2 prostate and colorectal cancers	
		2 prostate, colorectal and bladder cancers	
		2 endometrial cancers	
		2 ovarian and colorectal cancers	
		1 pancreatic cancer	
		1 gastric cancer	

**Table 2:** Results of mutational analysis conducted in family members of 34HBOC-mutated patients, with all pathogenic variants identified. The names of mutations are reported in bold.

Family	Gene	Mutation	Exon	Family Members (Diagnosis)	Age	Mutational Analysis
1	*BRCA1*	**c.117_118del (p.Cys39_Asp40delinsTer)**	3	Proband (Ovarian Cancer)	65	Mutated
				Daughter (Unaffected)	41	Wild Type
2	*BRCA1*	**c.181T > G (p.Cys61Gly)**	5	Proband (Breast Cancer)	30	Mutated
				Father (Prostate Cancer)	52	Mutated
				Sister (Unaffected)	31	Wild Type
				Sister (Unaffected)	27	Wild Type
				Sister (Unaffected)	21	Wild Type
3	*BRCA1*	**c.213-11T > G (IVS5-11T > G)**	6	Proband (Ovarian Cancer)	55	Mutated
				Son (Unaffected)	28	Wild Type
				Daughter (Unaffected)	24	Mutated
				Sister (Unaffected)	59	Mutated
4	*BRCA1*	**c.547 + 2T > A (IVS8 + 2T > A)**	8	Proband (Breast Cancer)	52	Mutated
				Mother (Breast Cancer)	45 ^†^	Mutated
				Aunt (Prostate Cancer)	60 ^†^	Mutated
				Aunt (Breast Cancer)	48 ^†^	Mutated
5	*BRCA1*	**c.798_799delTT (p.Ser267Lysfs)**	11	Proband (Breast and Ovarian Cancer)	56	Mutated
				Son (Unaffected)	28	Mutated
				Sister (Unaffected)	65	Wild Type
				Brother (Unaffected)	61	Mutated
6	*BRCA1*	c.1796_1800del (p.Ser599*)	11	Proband (Ovarian Cancer)	58	Mutated
				Sister (Unaffected)	55	Wild Type
7	*BRCA1*	**c.3351dup (p.Gln1118fs)**	11	Proband (Breast Cancer)	50	Mutated
				Cousin (Prostate Cancer)	53	Mutated
8	*BRCA1*	**c.3607 C > T (p.Arg1203Ter)**	11	Proband (Breast Cancer)	40	Mutated
				Sister (Unaffected)	39	Mutated
				Niece (Unaffected)	18	Wild Type
9	*BRCA1*	**c.3756_3759delGTCT (p.Ser1253ArgfsTer10)**	11	Proband (Ovarian Cancer)	59	Mutated
				Son (Unaffected)	36	Wild Type
				Daughter (Unaffected)	39	Wild Type
10	*BRCA1*	**c.5123C > A** **(p.Ala1708Glu)**	18	Proband (Breast and Ovarian Cancer)	52	Mutated
				Sister (Unaffected)	54	Mutated
				Brother (Unaffected)	51	Wild Type
11	*BRCA1*	**c.5123C > A** **(p.Ala1708Glu)**	18	Proband (Ovarian Cancer)	52	Mutated
				Sister (Melanoma)	55	Mutated
				Sister (Unaffected)	47	Wild Type
				Cousin (Unaffected)	50	Wild Type
12	*BRCA1*	**c.5123C > A** **(p.Ala1708Glu)**	18	Proband (Colon Cancer)	54	Mutated
				Sister (Unaffected)	55	Mutated
				Sister (Unaffected)	59	Mutated
				Cousin (Colon Cancer)	54	Wild Type
13	*BRCA1*	**c.5266dupC** **(p.Gln1756Profs)**	20	Proband (Breast and Ovarian Cancer)	64	Mutated
				Daughter (Unaffected)	36	Wild Type
				Son (Unaffected)	38	Mutated
				Brother (Unaffected)	66	Mutated
				Niece (Breast Cancer)	32	Mutated
				Niece (Prostate Cancer)	41	Mutated
14	*BRCA1*	**c.5266dupC** **(p.Gln1756Profs)**	20	Proband (Breast Cancer)	51	Mutated
				Daughter (Unaffected)	28	Mutated
15	*BRCA1*	**c.5266dupC** **(p.Gln1756Profs)**	20	Proband (Ovarian Cancer)	43	Mutated
				Sister (Unaffected)	27	Wild Type
				Brother (Unaffected)	39	Mutated
				Cousin (Breast and Ovarian Cancer)	46	Mutated
				Cousin (Unaffected)	57	Wild Type
				Cousin (Unaffected)	54	Wild Type
				Cousin (Prostate Cancer)	50	Mutated
16	*BRCA1* *BRCA2*	**c.547 + 2T > A (IVS8 + 2T > A)** **c.2830A > T (p.Lys944Ter)**	8 11	Proband (Bilateral Breast Cancer)	32	Mutated
				Sister (Unaffected)	48	Wild Type
				Mother (Unaffected)	68	Wild Type
				Father (Unaffected)	78	Mutated
				Aunt (Unaffected)	74	Wild Type
				Cousin (Unaffected)	50	Wild Type
				Cousin (Breast Cancer)	52	Mutated
				Niece (Unaffected)	25	Mutated
				Nephew (Unaffected)	27	Mutated
				Nephew (Unaffected)	21	Mutated
17	*BRCA2*	**c.67 + 1G > A (IVS2 + 1G > A)**	2	Proband (Breast Cancer)	60	Mutated
				Son (Unaffected)	56	Mutated
				Daughter (Unaffected)	54	Wild Type
				Daughter (Unaffected)	51	Wild Type
18	*BRCA2*	**c.1238delT (p.Leu413HisfsX17)**	10	Proband (Breast Cancer)	65	Mutated
				Son (Unaffected)	42	Mutated
				Son (Unaffected)	38	Mutated
				Son (Unaffected)	37	Mutated
				Brother (Unaffected)	71	Mutated
				Nephew (Unaffected)	37	Wild Type
19	*BRCA2*	**c.1496_1497delAG (p.Gln499fs)**	10	Proband (Breast Cancer)	41	Mutated
				Son (Unaffected)	21	Mutated
				Daughter (Unaffected)	24	Mutated
				Sister (Breast Cancer)	55	Mutated
				Nephew (Unaffected)	24	Mutated
				Niece (Unaffected)	36	Mutated
				Niece (Unaffected)	35	Mutated
				Cousin (Prostate Cancer)	47	Mutated
				Cousin (Unaffected)	41	Wild Type
20	*BRCA2*	**c.4322_4323delAG (p.Glu1441ValfsTer3)**	11	Proband (Breast Cancer)	62	Mutated
				Father (Prostate Cancer)	73	Mutated
21	*BRCA2*	**c.5796_5797delTA (p.His1932Glnfs)**	11	Proband (Breast Cancer)	72	Mutated
				Cousin (Prostate Cancer)	72	Mutated
				Cousin (Breast Cancer)	68	Wild Type
22	*BRCA2*	**c.6037A > T** **(p.Lys2013Ter)**	11	Proband (Ovarian Cancer)	66	Mutated
				Daughter (Unaffected)	28	Mutated
23	*BRCA2*	**c.6450dup** **(p.Val2151fs)**	11	Proband (Breast Cancer)	54	Mutated
				Son (Unaffected)	24	Wild Type
				Daughter (Unaffected)	21	Mutated
24	*BRCA2*	**c.6468_6469delTC** **(p.Gln2157Ilefs)**	11	Proband (Ovarian and Colon Cancer)	62	Mutated
				Daughter (Unaffected)	40	Wild Type
25	*BRCA2*	**c.6468_6469delTC** **(p.Gln2157Ilefs)**	11	Proband (Breast Cancer)	49	Mutated
				Sister (Ovarian Cancer)	53	Mutated
26	*BRCA2*	**c.6486_6489delACAA** **(p.Lys2162Asnfs)**	11	Proband (Breast Cancer)	50	Mutated
				Daughter (Unaffected)	30	Mutated
				Daughter (Unaffected)	28	Mutated
				Sister (Unaffected)	52	Wild Type
				Brother (Unaffected)	49	Mutated
27	*BRCA2*	**c.7007G > A (p.Arg2336His)**	13	Proband (Breast Cancer)	32	Mutated
				Mother (Unaffected)	57	Wild type
				Father (Prostate Cancer)	63	Mutated
				Sister (Unaffected)	27	Wild type
28	*BRCA2*	**c.7857G > A** **(p.Trp2619Ter)**	17	Proband (Breast Cancer)	45	Mutated
				Sister (Unaffected)	48	Wild Type
29	*BRCA2*	**c.8954_8955delGTTinsAA**	23	Proband (Colon Cancer)	76	Mutated
				Son (Unaffected)	39	Wild Type
				Daughter (Unaffected)	44	Mutated
				Father (Breast Cancer)	76	Mutated
				Sister (Thyroid Cancer)	70	Mutated
				Brother (Prostate Cancer)	66	Mutated
				Niece (Unaffected)	44	Mutated
				Niece (Unaffected)	23	Wild type
				Nephew (Unaffected)	42	Wild type
				Nephew (Unaffected)	44	Mutated
30	*BRCA2* *BRCA2*	**c.631G > A** **(p.Val221Ile)** **c.7008-2A > T** **(IVS13-2A > T)**	7 14	Proband (Breast Cancer)	46	Mutated
				Daughter (Unaffected)	23	Mutated
				Daughter (Unaffected)	22	Mutated
				Daughter (Unaffected)	18	Wild Type
				Sister (Unaffected)	46	Mutated
				Sister (Unaffected)	42	Wild Type
				Sister (Unaffected)	36	Wild Type
31	*PALB2*	**c.1919C > A (p.Ser640Ter)**	5	Proband (Breast Cancer)	30	Mutated
				Father (Prostate Cancer)	70	Mutated
32	*MUTYH*	**c.1437_1439del GGA** **(p.Glu480del)**	14	Proband (Breast Cancer)	85	Mutated
				Brother (Prostate Cancer)	78	Mutated
				Daughter (Endometrial Cancer)	55	Mutated
				Son (Unaffected)	58	Mutated
33	*RAD51C*	**c.(705 + 1_706-1)_(837 + 1_838-1)del**	5	Proband (Gastric Cancer)	58	Mutated
				Cousin (Endometrial Cancer)	54	Mutated
34	*TP53*	**c.817C > T (p.Arg273Cys)**	8	Proband (Breast Cancer)	46	Mutated
				Daughter (Unaffected)	20	Mutated
				Son (Unaffected)	24	Mutated

†: dead.

## Data Availability

Not applicable.

## References

[1] Rebello R.J., Oing C., Knudsen K.E., Loeb S., Johnson D.C., Reiter R.E., Gillessen S., Van der Kwast T., Bristow R.G. (2021). Prostate cancer. Nat. Rev. Dis. Prim..

[2] Heidegger I., Tsaur I., Borgmann H., Surcel C., Kretschmer A., Mathieu R., De Visschere P., Valerio M., Bergh R.C.V.D., Ost P. (2019). EAU-YAU Prostate Cancer Working Party. Hereditary prostate cancer—Primetime for genetic testing?. Cancer Treat. Rev..

[3] Vietri M., D’Elia G., Caliendo G., Resse M., Casamassimi A., Passariello L., Albanese L., Cioffi M., Molinari A. (2021). Hereditary Prostate Cancer: Genes Related, Target Therapy and Prevention. Int. J. Mol. Sci..

[4] Rebbeck T.R. (2016). Prostate Cancer Genetics: Variation by Race, Ethnicity, and Geography. Semin. Radiat. Oncol..

[5] Barber L.E., Gerke T., Markt S.C., Peisch S.F., Wilson K.M., Ahearn T.U., Giovannucci E.L., Parmigiani G., Mucci L.A. (2018). Family History of Breast or Prostate Cancer and Prostate Cancer Risk. Clin. Cancer Res..

[6] Vietri M.T., D’Elia G., Caliendo G., Casamassimi A., Resse M., Passariello L., Cioffi M., Molinari A.M. (2020). Double mutation of APC and BRCA1 in an Italian family. Cancer Genet..

[7] Vietri M.T., D’Elia G., Caliendo G., Casamassimi A., Federico A., Passariello L., Cioffi M., Molinari A.M. (2021). Prevalence of mutations in BRCA and MMR genes in patients affected with hereditary endometrial cancer. Med. Oncol..

[8] Vietri M.T., Molinari A.M., De Paola M.L., Cantile F., Fasano M., Cioffi M. (2012). Identification of a novel in-frame deletion in *BRCA2* and analysis of variants of *BRCA1/2* in Italian patients affected with hereditary breast and ovarian cancer. Clin. Chem. Lab. Med. (CCLM).

[9] Vietri M.T., D’Elia G., Benincasa G., Ferraro G., Caliendo G., Nicoletti G.F., Napoli C. (2021). DNA methylation and breast cancer: A way forward (Review). Int. J. Oncol..

[10] Das S., Salami S.S., Spratt D.E., Kaffenberger S.D., Jacobs M.F., Morgan T.M. (2019). Bringing Prostate Cancer Germline Genetics into Clinical Practice. J. Urol..

[11] González-Santiago S., Cajal T.R.Y., Aguirre E., Alés-Martínez J.E., Andrés R., Balmaña J., Graña B., Herrero A., Llort G., the SEOM Hereditary Cancer Working Group (2019). SEOM clinical guidelines in hereditary breast and ovarian cancer (2019). Clin. Transl. Oncol..

[12] Vietri M.T., Molinari A.M., Caliendo G., De Paola M.L., D’Elia G., Gambardella A.L., Petronella P., Cioffi M. (2013). Double heterozygosity in the BRCA1 and BRCA2 genes in Italian family. Clin. Chem. Lab. Med..

[13] Vietri M.T., Caliendo G., Schiano C., Casamassimi A., Molinari A.M., Napoli C., Cioffi M. (2015). Analysis of PALB2 in a cohort of Italian breast cancer patients: Identification of a novel PALB2 truncating mutation. Fam. Cancer.

[14] Vietri M.T., Caliendo G., Casamassimi A., Cioffi M.L., De Paola M., Napoli C., Molinari A.M. (2015). A novel PALB2 truncating mutation in an Italian family with male breast cancer. Oncol. Rep..

[15] Plon S.E., Eccles D.M., Easton D., Foulkes W.D., Genuardi M., Greenblatt M.S., Hogervorst F.B., Hoogerbrugge N., Spurdle A.B., Tavtigian S.V. (2008). Sequence variant classification and reporting: Recommendations for improving the interpretation of cancer susceptibility genetic test results. Hum. Mutat..

[16] Pilarski R. (2019). The Role of *BRCA* Testing in Hereditary Pancreatic and Prostate Cancer Families. Am. Soc. Clin. Oncol. Educ. Book.

[17] Sokolova A.O., Cheng H.H. (2020). Genetic Testing in Prostate Cancer. Curr. Oncol. Rep..

[18] Zhen J.T., Syed J., Nguyen K.A., Leapman M.S., Agarwal N., Ms K.B., Llor X., Hofstatter E., Shuch B. (2018). Genetic testing for hereditary prostate cancer: Current status and limitations. Cancer.

[19] Pritchard C.C., Mateo J., Walsh M.F., De Sarkar N., Abida W., Beltran H., Garofalo A., Gulati R., Carreira S., Eeles R. (2016). Inherited DNA-Repair Gene Mutations in Men with Metastatic Prostate Cancer. N. Engl. J. Med..

[20] Gandaglia G., Briganti A., Montorsi F. (2021). Reimagining prostate cancer screening: The IMPACT of germline mutations. Lancet Oncol..

[21] Costa D., Scognamiglio M., Fiorito C., Benincasa G., Napoli C. (2019). Genetic background, epigenetic factors and dietary interventions which influence human longevity. Biogerontology.

[22] Sarno F., Benincasa G., List M., Barabasi A.-L., Baumbach J., Ciardiello F., Filetti S., Glass K., Loscalzo J., the International Network Medicine Consortium (2021). Clinical epigenetics settings for cancer and cardiovascular diseases: Real-life applications of network medicine at the bedside. Clin. Epigenetics.

[23] Maiorino M.I., Schisano B., Di Palo C., Vietri M.T., Cioffi M., Giugliano G., Giugliano D., Esposito K. (2010). Interleukin-20 circulating levels in obese women: Effect of weight loss. Nutr. Metab. Cardiovasc. Dis..

[24] Cioffi M., Riegler G., Vietri M.T., Pilla P., Caserta L., Carratù R., Sica V., Molinari A.M. (2004). Serum p53 antibodies in patients affected with ulcerative colitis. Inflamm. Bowel. Dis..

[25] Bellastella G., Maiorino M.I., De Bellis A., Vietri M.T., Mosca C., Scappaticcio L., Pasquali D., Esposito K., Giugliano D. (2016). Serum but not salivary cortisol levels are influenced by daily glycemic oscillations in type 2 diabetes. Endocrine.

[26] Pritzlaff M., Tian Y., Reineke P., Stuenkel A.J., Allen K., Gutierrez S., Jackson M., Dolinsky J.S., LaDuca H., Xu J. (2020). Diagnosing hereditary cancer predisposition in men with prostate cancer. Genet. Med..

[27] Vietri M.T., Caliendo G., D’Elia G., Resse M., Casamassimi A., Minucci P.B., Ioio C.D., Cioffi M., Molinari A.M. (2020). Five Italian Families with Two Mutations in *BRCA* Genes. Genes.

[28] Iversen E.S., Lipton G., Hart S.N., Lee K.Y., Hu C., Polley E.C., Pesaran T., Yussuf A., LaDuca H., Chao E. (2022). An integrative model for the comprehensive classification of BRCA1 and BRCA2 variants of uncertain clinical significance. npj Genom. Med..

[29] Lyra P.C.M., Nepomuceno T.C., de Souza M.L.M., Machado G.F., Veloso M.F., Henriques T.B., dos Santos D.Z., Ribeiro I.G., Ribeiro R.S., Rangel L.B.A. (2020). Integration of functional assay data results provides strong evidence for classification of hundreds of BRCA1 variants of uncertain significance. Genet. Med..

[30] Edwards S.M., Evans D.G., Hope Q., Norman A.R., Barbachano Y., Bullock S., Kote-Jarai Z., Meitz J., Falconer A., Osin P. (2010). UK Genetic Prostate Cancer Study Collaborators and BAUS Section of Oncology. Pros-tate cancer in BRCA2 germline mutation carriers is associated with poorer prognosis. Br. J. Cancer.

[31] Patel V.L., Busch E.L., Friebel T.M., Cronin A., Leslie G., McGuffog L., Adlard J., Agata S., Agnarsson B.A., Ahmed M. (2020). Association of Genomic Domains in *BRCA1* and *BRCA2* with Prostate Cancer Risk and Aggressiveness. Cancer Res..

[32] Paulo P., Pinto P., Peixoto A., Santos C., Pinto C., Rocha P., Veiga I., Soares G., Machado C., Ramos F. (2017). Validation of a Next-Generation Sequencing Pipeline for the Molecular Diagnosis of Multiple Inherited Cancer Predisposing Syndromes. J. Mol. Diagn..

[33] Horak P., Weischenfeldt J., von Amsberg G., Beyer B., Schütte A., Uhrig S., Gieldon L., Klink B., Feuerbach L., Hübschmann D. (2019). Response to olaparib in a *PALB2* germline mutated prostate cancer and genetic events associated with resistance. Mol. Case Stud..

[34] Liu Z., Guo H., Zhu Y., Xia Y., Cui J., Shi K., Fan Y., Shi B., Chen S. (2020). TP53 alterations of hormone-naïve prostate cancer in the Chinese population. Prostate Cancer Prostatic Dis..

[35] Teroerde M., Nientiedt C., Duensing A., Hohenfellner M., Stenzinger A., Duensing S., Bott S.R.J., Ng K.L. (2021). Revisiting the Role of p53 in Prostate Cancer. Prostate Cancer.

[36] D’Elia G., Caliendo G., Casamassimi A., Cioffi M., Molinari A.M., Vietri M.T. (2018). APC and MUTYH Analysis in FAP Patients: A Novel Mutation in APC Gene and Genotype-Phenotype Correlation. Genes.

[37] Vietri M.T., D’Elia G., Caliendo G., Albanese L., Signoriello G., Napoli C., Molinari A.M. (2022). Pancreatic Cancer with Mutation in BRCA1/2, MLH1, and APC Genes: Phenotype Correlation and Detection of a Novel Germline BRCA2 Mutation. Genes.

[38] Nicolosi P., Ledet E., Yang S., Michalski S., Freschi B., O’Leary E., Esplin E.D., Nussbaum R.L., Sartor O. (2019). Prevalence of Germline Variants in Prostate Cancer and Implications for Current Genetic Testing Guidelines. JAMA Oncol..

[39] Thalgott M., Kron M., Brath J.M., Ankerst D.P., Thompson I.M., Gschwend J.E., Herkommer K. (2017). Men with family history of prostate cancer have a higher risk of disease recurrence after radical prostatectomy. World J. Urol..

[40] Castro E., Goh C., Olmos D., Saunders E., Leongamornlert D., Tymrakiewicz M., Mahmud N., Dadaev T., Govindasami K., Guy M. (2013). Germline *BRCA* Mutations Are Associated with Higher Risk of Nodal Involvement, Distant Metastasis, and Poor Survival Outcomes in Prostate Cancer. J. Clin. Oncol..

[41] Castro E., Goh C., Leongamornlert D., Saunders E., Tymrakiewicz M., Dadaev T., Govindasami K., Guy M., Ellis S., Frost D. (2015). Effect of BRCA Mutations on Metastatic Relapse and Cause-specific Survival After Radical Treatment for Localised Prostate Cancer. Eur. Urol..

[42] Narod S.A., Neuhausen S., Vichodez G., Armel S., Lynch H.T., Ghadirian P., Cummings S., Olopade O., Stoppa-Lyonnet D., Couch F. (2008). Rapid progression of prostate cancer in men with a BRCA2 mutation. Br. J. Cancer.

[43] Ren Z.-J., Cao D.-H., Zhang Q., Ren P.-W., Liu L.-R., Wei Q., Wei W.-R., Dong Q. (2019). First-degree family history of breast cancer is associated with prostate cancer risk: A systematic review and meta-analysis. BMC Cancer.

[44] Vietri M.T., Caliendo G., D’Elia G., Resse M., Casamassimi A., Minucci P.B., Cioffi M., Molinari A.M. (2020). BRCA and PALB2 mutations in a cohort of male breast cancer with one bilateral case. Eur. J. Med Genet..

[45] Marabelle A., Le D.T., Ascierto P.A., Di Giacomo A.M., De Jesus-Acosta A., Delord J.-P., Geva R., Gottfried M., Penel N., Hansen A.R. (2020). Efficacy of Pembrolizumab in Patients with Noncolorectal High Microsatellite Instability/Mismatch Repair–Deficient Cancer: Results From the Phase II KEYNOTE-158 Study. J. Clin. Oncol..

[46] Montisci A., Vietri M.T., Palmieri V., Sala S., Donatelli F., Napoli C. (2021). Cardiac Toxicity Associated with Cancer Immunotherapy and Biological Drugs. Cancers.

[47] Montisci A., Palmieri V., Liu J.E., Vietri M.T., Cirri S., Donatelli F., Napoli C. (2021). Severe Cardiac Toxicity Induced by Cancer Therapies Requiring Intensive Care Unit Admission. Front. Cardiovasc. Med..

[48] Carreira S., Porta N., Arce-Gallego S., Seed G., Llop-Guevara A., Bianchini D., Rescigno P., Paschalis A., Bertan C., Baker C. (2021). Biomarkers Associating with PARP Inhibitor Benefit in Prostate Cancer in the TOPARP-B Trial. Cancer Discov..

